# Epigenetic silencing of *RASSF1A* deregulates cytoskeleton and promotes malignant behavior of adrenocortical carcinoma

**DOI:** 10.1186/1476-4598-12-87

**Published:** 2013-08-05

**Authors:** Reju Korah, James M Healy, John W Kunstman, Annabelle L Fonseca, Amir H Ameri, Manju L Prasad, Tobias Carling

**Affiliations:** 1 Department of Surgery, Yale Endocrine Neoplasia Laboratory, Yale University School of Medicine, 333 Cedar Street, TMP202, Box 208062, New Haven, CT 06520, USA; 2 Departments of Surgery, Yale University School of Medicine, New Haven, CT, USA; 3 Department of Pathology, Yale University School of Medicine, New Haven, CT, USA

**Keywords:** Adrenal cortex, Carcinoma, Adenoma, RASSF1A, Hypermethylation, Epigenetic silencing, Cytoskeleton

## Abstract

**Background:**

Adrenocortical carcinoma (ACC) is a rare endocrine malignancy with high mutational heterogeneity and a generally poor clinical outcome. Despite implicated roles of deregulated TP53, IGF-2 and Wnt signaling pathways, a clear genetic association or unique mutational link to the disease is still missing. Recent studies suggest a crucial role for epigenetic modifications in the genesis and/or progression of ACC. This study specifically evaluates the potential role of epigenetic silencing of *RASSF1A*, the most commonly silenced tumor suppressor gene, in adrenocortical malignancy.

**Results:**

Using adrenocortical tumor and normal tissue specimens, we show a significant reduction in expression of *RASSF1A* mRNA and protein in ACC. Methylation-sensitive and -dependent restriction enzyme based PCR assays revealed significant DNA hypermethylation of the *RASSF1A* promoter, suggesting an epigenetic mechanism for *RASSF1A* silencing in ACC. Conversely, the *RASSF1A* promoter methylation profile in benign adrenocortical adenomas (ACAs) was found to be very similar to that found in normal adrenal cortex. Enforced expression of ectopic *RASSF1A* in the SW-13 ACC cell line reduced the overall malignant behavior of the cells, which included impairment of invasion through the basement membrane, cell motility, and solitary cell survival and growth. On the other hand, expression of *RASSF1A/A133S*, a loss-of-function mutant form of *RASSF1A*, failed to elicit similar malignancy-suppressing responses in ACC cells. Moreover, association of RASSF1A with the cytoskeleton in *RASSF1A*-expressing ACC cells and normal adrenal cortex suggests a role for RASSF1A in modulating microtubule dynamics in the adrenal cortex, and thereby potentially blocking malignant progression.

**Conclusions:**

Downregulation of RASSF1A via promoter hypermethylation may play a role in the malignant progression of adrenocortical carcinoma possibly by abrogating differentiation-promoting *RASSF1A*- microtubule interactions.

## Background

Adrenocortical carcinoma (ACC) is a rare endocrine malignancy, with an annual incidence of approximately 0.5 - 2 cases per million [1,2]. Despite recent progress, including the first randomized controlled trial of conventional chemotherapy in ACC patients [3], these tumors remain a clinical challenge, with an overall 5-year survival of 16-38% even with aggressive surgical and oncologic therapy [4]. The major reasons for this include (a) an initially silent clinical course that ultimately manifests in advanced disease, with 30-40% of patients having metastatic disease upon initial diagnosis [5,6] and, (b) the incomplete understanding of the molecular pathogenesis of the disease.

Several well-known tumor suppressor- and oncogene-signaling pathways have been previously described as implicated in ACC tumorigenesis. In addition to somatic mutations in exons 5–8 and 2–11, germ-line variants in TP53 also has been reported in sporadic ACCs [7-11]. Moreover, the Wnt signaling pathway is found frequently altered in ACC, with abnormal accumulation of β-catenin present in 85% of ACC, and somatic activating mutations present in 30% of ACCs [12]. In patients with Carney Complex and Isolated Primary Pigmented Nodular Adrenocortical Disease, somatic inactivating mutations in *PRKAR1A* have been associated with ACC [13]. Although controversial, deregulated IGF signaling also has been implicated in the origin and/or progression of ACC [14].

The Ras-Association Domain Family 1A (RASSF1A) protein is a 37kDa ubiquitously expressed isoform of the *RASSF1* gene with demonstrated tumor suppressor function in a variety of tissues [15-17]. *RASSF1* is expressed as multiple splice variants, with each containing an RA domain, a C-terminal SARAH protein-protein interaction motif, a phosphorylation site for the DNA repair kinase ATM, and a cysteine-rich domain homologous to the Raf-1 diacylglycerol binding domain [15]. The *RASSF1* gene has two associated CpG islands (CpG islands A & C), with the smaller 737 bp-CpG island A spanning the promoter region for *RASSF1A* while CpG island C spans exon 2 that encompasses promoter regions for isoforms RASFF1B and RASFF1C [18].

Multiple studies have suggested a variety of roles for *RASSF1A* in suppressing carcinogenesis. RASSF1A restricts unscheduled proliferation, survival, and migration signaling downstream of a variety of oncogenes, including *RAS* and *BRAF*[19,20]. *RASSF1A* can regulate cell proliferation via protein-protein interaction with *RAS*[21], can stabilize microtubule formation via a domain near the ATM phosphorylation target (S131) [22-25], and has demonstrated pro-apoptotic effects downstream to multiple pathways including *Hippo*, *SAPK-JNK*, and *MST1/MST2*[26]. *RASSF1A* can also suppress *K-Ras* and TNF-alpha induced resistance to apoptosis [22,25,27,28]. Furthermore, *RASSF1A* induction has been shown to suppress anchorage-dependent colony formation in non-squamous cell lung cancer cell-lines [29] and *RASSF1A* knockout mice have increased susceptibility to spontaneous tumor development [30]. However, its precise role and mechanism of action in most tumor types remains to be further clarified [31-34].

Epigenetic aberrations, including DNA methylation and histone modifications, are increasingly being recognized for their role in altering patterns of gene expression, potentially contributing to tumorigenesis [35]. Global DNA hypomethylation has been demonstrated in ACC [36,37] with locus-specific patterns of hypermethylation [36,38]. Genome-wide studies of the DNA methylomes of ACC and ACA have identified multiple genes with differential DNA methylation patterns; notably, including several tumor suppressor genes [38,39].

Hypermethylation of the *RASSF1* promoter responsible for RASSF1A expression has a well-established role in tumor progression in several organ systems and tissue types [26,40-45], including several endocrine tumors. Specifically, epigenetic suppression of *RASSF1A* expression in papillary thyroid carcinoma has been strongly implicated in early tumor formation [31,32,46,47]. Similarly, epigenetic silencing of *RASSF1A* has been demonstrated in neural crest tumors such as neuroblastoma and pheochromocytoma [46]. Alternatively, genetic silencing of *RASSF1A* gene by mutations and other aberrations are possible, but rarely seen in human cancers [47]. In this study, we hypothesized that RASSF1A functions as a tumor suppressor in adrenal cortex and that its epigenetic suppression by promoter methylation may be a key step in tumor progression. We also evaluated whether *RASSF1A* suppression in ACC is correlated with a more malignant phenotype. Furthermore, we investigated the functional consequence of reversing this suppression in an adrenocortical cell culture system with the aim towards understanding the mechanism of RASSF1A function in the adrenal cortex.

## Results

### Increased hypermethylation of CpG island A of the *RASSF1* promoter in adrenocortical carcinoma

**Figure 1:**
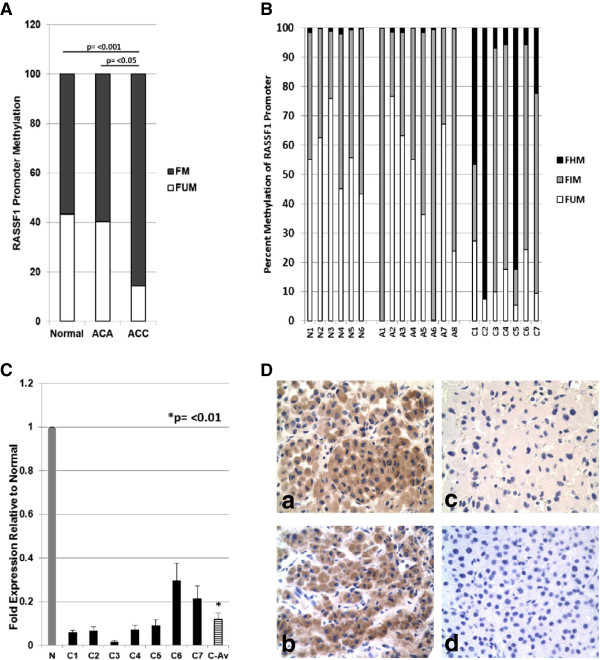
**RASSF1A expression and regulation in adrenal tumor. (A)** Averages of percentage methylated (FM) and unmethylated (FUM) CpGs in CpG island A of *RASSF1A* promoters in Normal adrenal cortex (n = 6), ACA (n = 8), and ACC (n = 7) samples are shown. FM includes both Hypermethylated (FHM) and intermediate methylated (FIM) fractions. **(B)** Methylation profiles of individual fresh-frozen normal adrenal cortex (N1 – N6), 8 ACAs (A1 – A8) and 7 ACC samples (C1 – C7) as determined by Epitect methyl II PCR assay. **(C)** Expression of *RASSF1A* mRNA determined by real-time qPCR in 7 ACC samples (C1 – C7) compared to the average expression in 6 normal samples (N) normalized to a value of 1.0. RASSF1A expressions in individual samples were also normalized to the average mRNA expression of house-keeping genes beta-actin (*Actb*) and TATA-binding protein (*TBP*). C-Av represents the average expression of all ACC samples. Data shown is from one of triplicate experiments that yielded similar results (mean ± SD). Independent sample t-test used to derive the p value (p = <0.01). **(D)** RASSF1A protein expression in normal **(a** &**b)** and ACC **(c** &**d)** FFPE tissue specimens demonstrated by immunohistochemistry through DAB staining (brown indicates RASSF1A protein expression) followed by nuclear counterstaining by hematoxylin (blue).

*RASSF1* CpG island A hypermethylation is the most common epigenetic mechanism observed in tumors with silenced RASSF1A function [31,32,46,47]. To test whether promoter hypermethylation and consequent *RASSF1A* silencing contributes to adrenocortical tumorigenesis, we first determined the methylation status of CpG island A of *RASSF1* in fresh-frozen ACC (n = 7), ACA (adrenocortical adenoma; n = 8) and normal adrenal cortex (n = 6) tissue specimens. Rarity of the disease and scarcity of adequate amounts of the specimens for assays constrained us from recruiting a larger cohort. We used a methylation-sensitive and -dependent restriction digestion based qPCR strategy to evaluate the methylation status of the 737bp area that spans *RASSF1* CpG island A. This technique enables qualitative characterization (i.e. – regions demonstrating hypo-/intermediate-/hyper- methylation) of DNA methylation. The overall methylation profiles of normal and benign ACA samples were found to be very similar (57% and 60% respectively) while malignant ACCs showed *a distinct* statistically significant increase (86%) in the methylated fraction (Figure 1A). Analysis of methylation patterns in individual samples showed very low levels of hypermethylation (which represents >60% digestion by methylation-dependent restriction enzyme) in all normal and ACA cases (Figure 1B). Hypermethylation in normal adrenal cortex samples ranged from 0.2 – 2.0 % with an average of 0.35% and ACA samples ranged from 0.03 – 1.7% with an average hypermethylation of 0.7%. Conversely, all the ACC samples tested showed hypermethylation in excess of the maximum level observed in normal and ACA tissues. About 60% (4/7) of ACC samples had very high (>20%) hypermethylation of the CpG island A of the *RASSF1* promoter (Figure 1B).

### Reduced expression of RASSF1A in adrenocortical carcinoma (ACC)

A direct correlation between *RASSF1* promoter hypermethylation and reduced RASSF1A expression is observed in a variety of cancers (15). To test whether the observed CpG Island A hypermethylation in ACC (Figure 1B) is associated with a corresponding reduction in RASSF1A expression, we compared the mRNA and protein expression in ACC samples to that of normal adrenal cortex tissue samples. Quantitative PCR analysis of gene expression showed significantly decreased expression of *RASSF1A* mRNA in ACC samples (*P* < 0.01) ranging from 1% to 24% of average RASSF1A expression in normal adrenal cortex (Figure 1C). All ACC samples tested showed reduced expression of RASSF1A, irrespective of their clinical characteristics or malignant stages status (Table 1; Figure 1C). We also assessed the expression levels of RASSF1A protein in ACC samples by immunohistochemical staining. Histopathologically normal specimens (Figure 1D; a & b) showed markedly higher RASSF1A expression, while RASSF1A was undetectable in areas dominated by malignant cells (Figure 1D; c & d), suggesting a correlation between low mRNA expression and undetectable RASSF1A protein levels in ACC samples.

**Table 1:** Clinicopathological characteristics of patients

**ID**	**Age**	**Gender**	**ENSAT 2008 Stage**	**Metastasis**	**Diameter**	**Hormonal profile**	**Recurrence**
**C1**	**58**	**F**	**III**	**N**	**13 cm**	**Non-functional**	**Y**
**C2**	**32**	**F**	**III**	**N**	**12 cm**	**Androgen-producing**	**N**
**C3**	**62**	**F**	**III**	**Y**	**14 cm**	**Cortisol-producing**	**Y**
**C4**	**48**	**M**	**III**	**N**	**9 cm**	**Non-functional**	**N**
**C5**	**48**	**F**	**III**	**N**	**13 cm**	**Cortisol-producing**	**Y**
**C6**	**55**	**F**	**IV**	**Y**	**5.5 cm**	**Cortisol-producing**	**Y**
**C7**	**60**	**F**	**IV**	**Y**	**7.8 cm**	**Cortisol-producing**	**Y**

Diverse clinical and pathological characteristics of adrenocortical cancer (n = 7) patients selected for the study, is shown. ENSAT 2008 Staging: European Network for the Study of Endocrine Tumors; Y = Yes, N = No, F = Female, M = Male.

**Figure 2:**
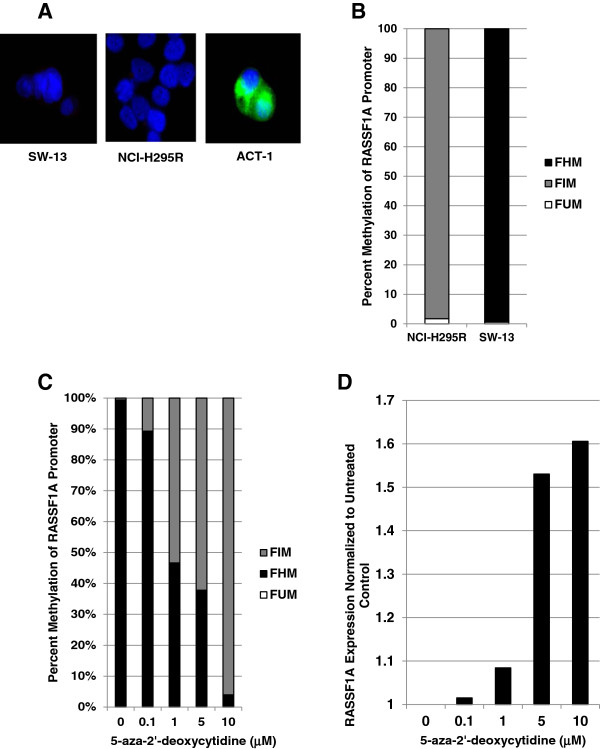
**RASSF1A expression and promoter methylation in ACC cell lines. (A)** Indirect immunofluorescence detection of RASSF1A protein (FITC – green) expression in SW-13, NCI-H295R and ACT-1 (a thyroid cancer cell line used as a positive control for RASSF1A expression) cells. Cell nuclei fluoresces blue due to DAPI fluorescence. **(B)** RASSF1A promoter methylation pattern in exponentially growing cultures of NCI-H295R and SW-13 cells as determined by Epitect methyl II PCR assay. Averages of percentage Hypermethylated (FHM) intermediate methylated (FIM), and unmethylated (FUM) CpGs are shown. **(C** &**D)** SW-13 cells were grown in the presence of varying (0, 0.1, 1, 5 and 10 μM) concentrations of 5-aza-2’-deoxycitidine for 48 hours and **(C)** Epitect methyl II assay was performed on genomic DNA to determine *RASSF1A* promoter methylation, and **(D) ***RASSF1A* mRNA expression was assayed by real-time qPCR. Average mRNA expression values of house-keeping genes beta-actin (*Actb*) and TATA-binding protein (*TBP*) were used for normalization.

To investigate the functional significance of promoter hypermethylation and consequent RASSF1A silencing in adrenocortical carcinogenesis, we sought to utilize a cell culture model. To identify a suitable model, we analyzed the RASSF1A expression pattern in two widely used ACC cell lines NCI-H295R and SW-13, by indirect immunofluorescence (Figure 2A). As shown in Figure 2A, both ACC cell lines revealed undetectable levels of RASSF1A, when compared with the expression in a thyroid cancer cell line ACT-1. Next, we examined the methylation pattern of NCI-H295R and SW-13 cells which showed very high levels of methylation in both cell types (Figure 2B). However, the methylation pattern appeared to be different between the two ACC cell lines. While NCI-H295R cells showed no hypermethylation, similar to ACA and normal adrenal tissue methylation, SW-13 cells showed more than 99% hypermethylation in the RASSF1 promoter (Figure 2B), similar to the hypermethylation levels observed in some ACC samples (note Figure 1B). Therefore, we chose SW-13 cells for further functional studies. To confirm *RASSF1A* promoter hypermethylation as the cause of RASSF1A downregulation in SW-13 cells, we treated the cells with a widely used de-methylating agent 5-aza-2’-deoxycytidine [35]. After 48 hours of treatment with 5-aza-2’-deoxycytidine, *RASSF1A* promoter analysis showed a dose-dependent reversal of hypermethylation (Figure 2C) and a consequent dose-dependent increase in the expression levels of *RASSF1A* mRNA (Figure 2D).

### Enforced expression of RASSF1A in SW-13 cells

**Figure 3:**
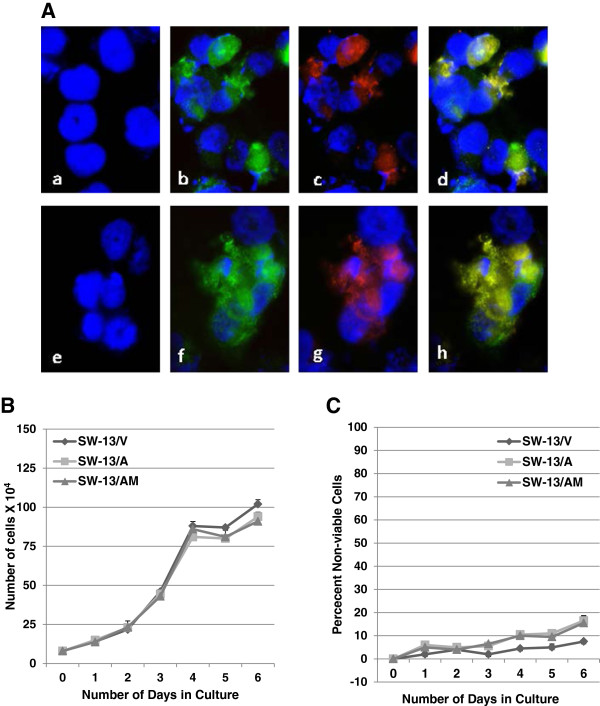
**Enforced expression of RASSF1A in ACC cells. (A)** SW-13 ACC cells that do not express RASSF1A **(a)** were transfected with empty **(a** &**e)**, *RASSF1A***(b** - **d)** or *RASSF1A/A13*3S **(f** - **h)** expression vectors. Expression of *RASSF1A* was determined by immunofluorescence detection of RASSF1A protein **(b** &**f)** or DDK tag **(c** &**g)** using anti-RASSF1 goat polyclonal and anti-DDK mAb respectively followed by anti-goat-FITC and anti-moue-TR secondary antibodies and DAPI for nuclear staining. Note co-localization of RASSF1A and DDK antigens **(d** &**h)** and absence of both in **a** &**e**. **(B** &**C)** Transient transfection was carried out in 6-well plates with a starting density of 80,000 cells/well and allowed to grow for 6 days to test the effect of RASSF1A and RASSF1A/A133S expression on growth potential **(B)** and survival **(C)** of SW-13 cells. Graphs represent one of 3 independent experiments that yielded similar results.

To test whether *RASSF1A* silencing contributes to the malignant progression of ACC, we re-expressed RASSF1A in SW-13 ACC cells that did not express detectable levels of RASSF1A (Figure 2A). In addition to the empty vector transfection controls (Figure 3A; a, e), we also expressed a variant of RASSF1A (RASSF1A/A133S) with demonstrated inability to elicit tumor suppressor function [48], as a control. Expression of ectopic RASSF1A (Figure 3A; b-d) and RASSF1A/A133S (Figure 3A; f-h) were confirmed using anti-RASSF1A antibody (Figure 3A; b & f) or anti-DDK antibody (Figure 3A; c & g), both of which co-localized to the same epitope (Figure 3A; d & h). After confirming the transfection efficiency to be in excess of 70% (Figure 3A) one day post-transfection, we determined the cell proliferation efficiency and cell survival for a period of 6 days post-transfection. Constitutive expression of RASSF1A (SW-13/A) or RASSF1A/A133S mutant (SW-13/AM) showed no significant impact on the proliferation (Figure 3B) or viability (Figure 3C) of SW-13 cells in comparison with SW-13 transiently transfected with the empty vector (SW-13/V) alone (Figures 3B &3C).

**Figure 4:**
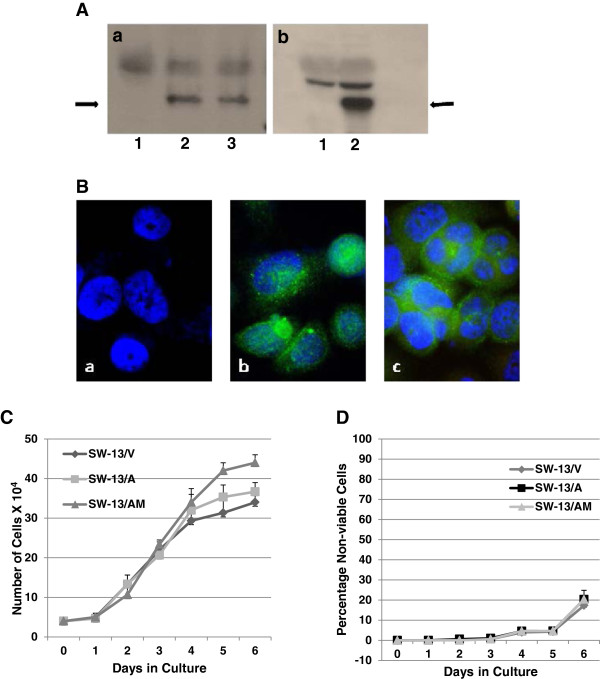
**Stable expression and selection of RASSF1A-expressing ACC cell lines.** SW-13 ACC cells were transfected with CMV-promoted *RASSF1A* and *RASSF1A/A133S* mutant genes and G-418 resistant clones were selected under low-seeding densities. Multiple clones were pooled to make populations to avoid variability in expression levels. **(A)** Western Immunoblot detection of RASSF1A and RASSF1A/A133S (arrows point to the RASSF1A bands) expression using anti-DDK **(a)** or anti-RASSF1A **(b)** antibody (lanes a1: transfected with empty vector; lane a2: RASSF1A; lane a3: RASSF1A/A133S; b1 empty vector, and lane b2 RASSF1A expression vectors). **(B)** Immunofluorescence detection of RASSF1A expression in **(a)** SW-13/N, **(b)** SW-13/A, and **(c)** SW-13/M cells, using anti-RASSF1A mAb followed by anti-mouse-FITC antibody (green). DAPI stained nucleus appears blue. **(C** &**D)** Established populations were grown for 7 days to determine the effect of constitutive expression of RASSF1A or RASSF1A/A133S on proliferation **(C)** and survival **(D)** in comparison to vector-transfected and selected cells. Graphs represent data from one of two independent experiments with similar results.

The tumor suppressor function of RASSF1A is context-dependent and is elicited via multiple and alternate signaling events such as pro-apoptotic, cell cycle arrest, mitotic arrest and/or cytoskeletal modifications [15]. After confirming the lack of apoptosis-inducing and cell cycle arrest functions in SW-13 cells via transient transfection experiments (Figures 3B &3C), we generated stable SW-13 cell derivatives that expressed RASSF1A and RASSF1A/A133S mutant proteins, to study potential tumor suppressor functions of RASSF1A in adrenal carcinomas. Expression of RASSF1A (SW-13/A) and RASSF1A/A133S (SW-13/AM) was verified following neomycin selection and subsequent population expansion, using Western immunoblots (Figure 4A; lanes a2, a3 & b2) and immunofluorescence (Figure 4B). Wild-type RASSF1A were found distributed both in the cytoplasm and nucleus of cells (Figure 4B; b) while RASSF1A/A133S mutant proteins were found predominantly localized in the cytoplasm (Figure 4B; c). Note lack of expression of endogenous RASSF1A in SW-13/V cells (Figure 4A: lane b1 and Figure 4B; a). Similar to the observation in transiently transfected cells (Figure 3B &3C), no significant detrimental effects on cell viability or growth were observed following stable expression in SW-13/V, SW-13/A, or SW-13/AM populations (Figure 4C &4D), suggesting alternate roles for RASSF1A silencing in ACC and possibly in SW-13 cell behavior.

### Re-expression of RASSF1A reduces malignant behavior of SW-13 cells

**Figure 5:**
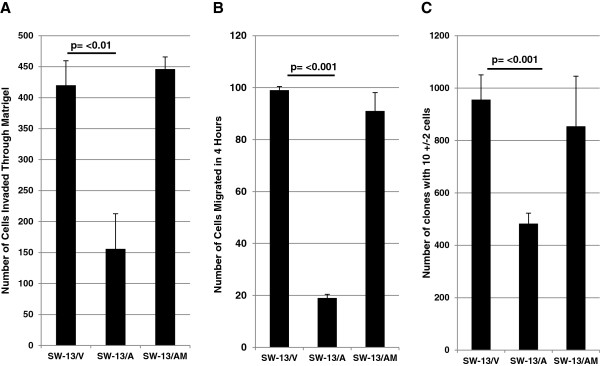
**Constitutive expression of RASSF1A reduces the invasive, migratory and clonogenic potentials of SW-13 cells. (A)**, SW-13/A and SW-13/AM cells were allowed to invade through Matrigel from upper chambers containing serum-free medium to lower chambers containing 10% FBS medium. After 24 hours, *and* invaded cells were fixed, stained with crystal violet and tabulated. Data represent results from one of two independent experiments with similar results. **(B)** SW-13/V, SW-13/A and SW-13/AM cells were allowed to migrate through modified Boyden Chambers (8 μM pore size) for 4 hours and migrated cells to the lower side of the membrane were fixed, stained with crystal violet and tabulated. Data from a representative experiment of triplicate experiments with similar results are shown. **(C)** SW-13/V, SW-13/A and SW-13/AM cells were seeded in 6-well plates in low densities (5000 cells/well) and allowed to grow for 7 days in G-418 containing medium. Cells were washed with PBS, fixed in 3.7 % formaldehyde solution stained with crystal violet and colonies with 10 +/− 2 cells were counted and averaged from 6 wells. Data from a representative experiment of quadruplicate experiments with similar results are shown.

Advanced features of malignancy, such as invasion of or migration to adjacent tissues, degradation of the extra cellular matrix (ECM), and clonogenic survival and growth, are hallmarks of aggressive tumors such as ACC that portend a poor clinical outcome. We evaluated whether constitutive expression of RASSF1A have any effect on the malignant phenotype of SW-13 cells. Cell invasiveness was evaluated using an overnight Matrigel invasion assay, which showed a significant reduction in the invasive potential of SW-13 cells constitutively expressing RASSF1A (SW-13/A) compared to empty vector (SW-13/V) or RASSF1A mutant (SW-13/AM) (Figure 5A). To test whether the reduced invasive potential is through an impaired migratory response, cells were allowed to migrate through 8μm pore-carrying cell culture inserts following a nutrient gradient. After 4 hours, SW-13 cells expressing *RASSF1A* showed a 5-fold reduction in the number of cells migrated across the membrane (Figure 5B), suggesting a strong motility-inhibitory response from re-expressed RASSF1A. Similarly, cells constitutively expressing high levels of RASSF1A also showed a significantly impaired clonogenic survival/growth response when compared to cells not expressing RASSF1A or cells expressing high levels of RASSF1A/A133S mutant proteins (Figure 5C). In summary, RASSF1A expression resulted in an overall reduced malignant behavior of SW-13 ACC cells which was not observed in cells expressing the RASSF1A/A133S mutant protein.

### RASSF1A alters malignant behavior of ACC cells by modulating microtubule organization

**Figure 6:**
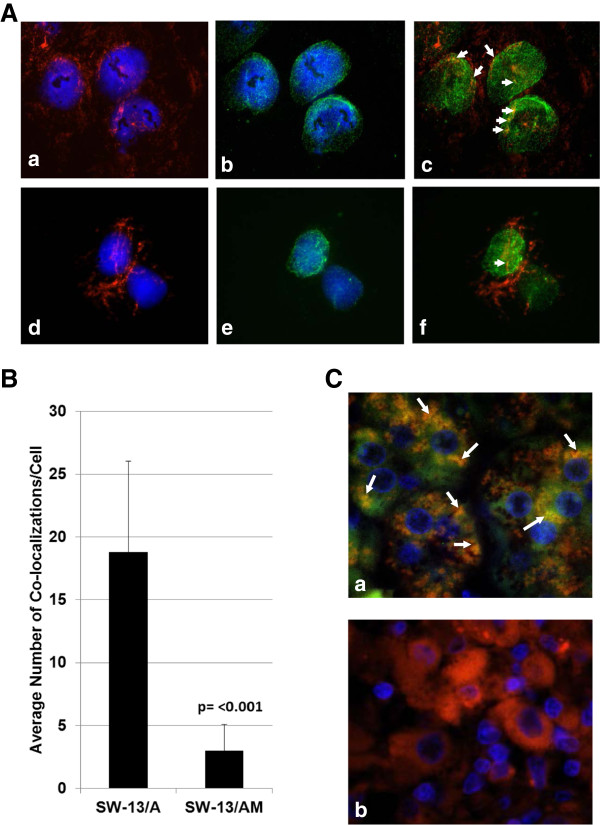
**Co-localization of RASSF1A with microtubules. (A)** SW-13/A **(a**, **b** &**c)** or SW-13/AM **(d**, **e** &**f)** cells were grown on glass cover slips in medium containing 400 ug/ml G418 and after 24 hours, cells were fixed in cold Acetone-Methanol (1:1) for 10 minutes followed by immunofluorescence detection of cytoskeleton (using Rhodamine-Phalloidin; **a** &**d**) or RASSF1A (using anti-RASSF1A antibody and FITC-conjugated secondary antibody; **b** &**e**) or both **(c** &**f)**. Cell nuclei fluoresces blue with DAPI. Arrows indicate areas of co-localization of RASSF1A and cytoskeleton **(c** &**f)**. **(B)** RASSF1A-microtubule co-localization points in comparable number of photomicrographs of SW-13/A and SW-13/AM cells representing multiple views from duplicate experiments were manually counted, tabulated and presented as a graph. **(C)** Representative photomicrographs showing indirect immunofluorescence detection of RASSF1A and microtubules in the normal adrenal cortex **(a)** and ACC **(b)** tissue specimens. Red fluorescence represents microtubules, green RASSF1A and blue DAPI-stained nuclei. Arrows indicate co-localization of RASSF1A and microtubules. (Total magnification: 1000X).

To test whether the observed malignant-dampening effect of RASSF1A in SW-13 cells is through modulating cytoskeletal function, we examined the localization pattern of RASSF1A (Figure 6A; b & c)) and RASSF1A/A133S mutant (Figure 6A; e & f) proteins in the context of localization of microtubule-binding phalloidins (Figure 6A; a, c, d & f). Co-localization of microtubules with RASSF1A was observed predominantly in cells expressing the wild-type RASSF1A protein (Note the arrows in Figure 6A; c), which was found significantly reduced (6B) in cells expressing RASSF1A/A133S mutant proteins (Figure 6A; f), suggesting a potential microtubule modulatory role for RASSF1A, not the mutant A133S mutant, in eliciting the observed reduced malignant behavior in SW-13 cells constitutively expressing RASSF1A. Despite the absence of RASSF1A/A133S co-localization with microtubules, the overall microtubule distribution appeared to be similar between RASSF1A-expressing and A133S mutant-expressing cells (6A; a & b). We also observed a similar co-localization pattern for RASSF1A and microtubules (Figure 6C; a) in normal adrenal cortex where microtubules appeared to have a punctate co-localization pattern of distribution with RASSF1A, in comparison to a more dispersed distribution found in ACC specimens that lack RASSF1A expression (Figure 6C; b). Although indirect, the co-localization of RASSF1A with microtubules both in normal adrenal cortex and ACC cells with reduced malignant properties (SW-13/A) suggests an anti-motility role for RASSF1A in adrenocortical carcinogenesis.

## Discussion

Neoplasias of the adrenal cortex present unique challenges in diagnosis and treatment, largely due to an incomplete understanding of the molecular pathogenesis of the disease. In this study, we examine the role of RASSF1A, a well-known tumor suppressor that has demonstrated roles in numerous other malignancies including several endocrine cancers, in adrenocortical carcinogenesis. Recent studies in gene expression profiling have suggested a potential role for aberrant DNA methylation events (both hypo- and hypermethylation) in the origin and/or progression of ACC, as in many other malignancies, including endocrine tumors such as neuroblastoma and pheochromocytoma [35-38,46,49,50]. RASSF1A, the most frequently silenced tumor suppressor via promoter methylation [15], thus is an attractive candidate to explore in the context of adrenocortical tumorigenesis.

Interrogation of the CpG Island A of the *RASSF1* promoter using the methyl screen technology showed a markedly increased hypermethylation pattern in ACC tissue samples. The promoter hypermethylation pattern observed in ACC was distinctly different from both normal adrenal cortex samples and benign adenomas, suggesting RASSF1A silencing as a possible later event in the overall adrenocortical tumorigenesis process. Expression analysis of RASSF1A in ACC demonstrated the functional consequence of hypermethylation as a significant decrease in both gene transcription and translation of *RASSF1A* in ACC cells as determined by qPCR, and immunohistochemistry respectively.

To test the hypothesized role for RASSF1A silencing in promoting adrenocortical malignancy, we sought to use a well-established cell culture model. We chose SW-13 ACC cell line that showed a comparable *RASSF1* promoter CpG Island A hypermethylation and undetectable RASSF1A protein expression. As the tumor suppressor function of RASSF1A manifests in promotion of apoptosis and downregulation of cell proliferation, viability and proliferation of SW-13 cells was first assessed following transient transfection with *RASSF1A*-expressing vector and found to be unaffected. Lack of promotion of cell death or cell cycle arrest by RASSF1A re-expression allowed us to generate SW-13 cell derivatives constitutively expressing RASSF1A. After confirming no growth disadvantage consequent to enforced over expression of RASSF1A, SW-13 cell variants constitutively expressing *RASSF1A* and *RASSF1A/A133S* were then assayed for other advanced malignancy or metastasis associated tumor behaviors such as invasion and solitary cell growth. Using a reconstituted basement membrane (Matrigel) invasion assay, a significant reduction in invasiveness was observed in SW-13 cells with forced RASSF1A expression (SW-13/A). To test whether the reduced invasive potential we observed in SW-13 cells with forced RASSF1A expression was due to decreased migratory ability, transwell migration assays were performed. The results mirrored the invasion assay, demonstrating significantly lower migratory potential with forced wild-type RASSF1A expression that was absent in SW-13/AM. The RASSF1A-mediated inhibition of the migratory response appeared to be sufficient to account for the observed anti-invasive effect and therefore, we did not investigate potential involvement of matrix metalloproteinases (MMPs) or their inhibitors in mediating the observed Matrigel invasion-inhibitory response. Finally, under low seeding conditions that mimic solitary cell growth, constitutive expression of RASSF1A reduced the clonogenicity of solitary SW-13 cells with decreased ability to establish, survive and grow into individual clones. The solitary growth inhibitory effect was not found in RASSF1A/A133S-expressing SW-13 cells.

Changes in microtubule dynamics are essential events in initial local tumor invasion as well as later metastatic spread. RASSF1A has demonstrated ability in influencing cytoskeletal dynamics by physically binding to filamentous actin as well as inhibiting tubulin polymerization [23,24,28,51,52]. To test whether ectopically-expressed RASSF1A physically interacts with microtubules, we treated cells with microtubule-binding phalloidin along with RASSF1A-detecting antibodies. In cells expressing RASSF1A, but not in cells expressing the A133S mutant, we observed sporadic co-localization of RASSF1A with microtubules, which may have a stabilizing effect on microtubule dynamics. Interestingly, it has been recently suggested that the A133S point mutation in RASSF1A abrogates its ability to modulate cytoskeletal interactions, contributing to loss of its tumor suppressor function [53]. Stabilization of microtubules by RASSF1A has been shown to disrupt malignant behavior in many cancer cell types [54]. Although RASSF1A-microtubule co-localization was not ubiquitously observed throughout the cell, the detected limited interaction may lead to partial global disruption of microtubule dynamics and contribute to the observed dampening of malignant behaviors in SW-13 cells. To assess whether the implicated cytoskeleton-stabilizing role is absent in ACC tissue, we compared the general organization of cytoskeleton in ACC tissue to that of normal adrenal cortex. In ACC, the cytoskeleton appeared to be very diffuse while in the normal cortex, the cytoskeleton co-localized with RASSF1A into a pattern of punctuated structures (Figure 6C), suggestive of a more stabilized organization. However, more experiments are needed to confirm the functional significance of such predicted RASSF1A-cytoskeletal interactions.

In summary, the results of this study strongly suggest functional evidence of a potential oncosuppressor role for RASSF1A in adrenocortical carcinogenesis. Although implicated to play a cytoskeleton-modulating role in other tissues, this study provides the first evidence for a cytoskeleton-stabilizing role for RASSF1A in adrenal cortex. Whether silencing of RASSF1A serves as a driving event driving benign ACAs to malignant ACC status, need further investigation.

## Materials and methods

### Tissue acquisition

Informed consent was obtained from patients prior to surgical resection of adrenal tissue according to a protocol approved by the local Institutional Review Board and Yale Pathology Tissue services. Tissue was flash-frozen in liquid nitrogen and stored at -80°C until processed for study. Specimens displaying unequivocal histopathological characteristics of ACC (n = 7), ACA (n = 8), and normal adrenal cortical tissue (n = 6) samples were selected for use in the study. Consecutive unstained and Hematoxylin & Eosin (H&E) stained 5 μm sections of tumor and normal formalin-fixed paraffin embedded (FFPE) tissue samples were obtained from Yale Tissue Pathology services. All samples were evaluated by experienced endocrine pathologists before processing.

### DNA, RNA, and Protein preparation

Genomic DNA from tissue samples were isolated using the DNeasy blood and tissue kit from Qiagen (Valencia, CA). Total RNA from the samples were isolated using the RNeasy Mini Kit (Qiagen, Valencia, CA,) after rotor-stator homogenization, per the manufacturer’s recommendations. Quantity and quality of prepared DNA and RNA was assessed by spectrophotometry (NanoDrop Technologies, Inc., Thermo Fischer, Waltham, MA) and 1% agarose gel electrophoresis. Total protein from cultured cells were extracted using Laemmli buffer (BioRad, Hercules, CA) and protein concentrations were measured using the Pierce BCA Protein assay Kit (Thermo Scientific, Rockford, IL) and Multimax detection system (Promega, Madison, WI), per the manufacturer’s instructions.

### Gene expression analysis

Total RNA (100 ng) was reverse transcribed using Superscript III reverse transcriptase (Applied Biosystems, Rockville, MD). Quantitative real-time PCR (qPCR) was performed on triplicate samples using TaqMan PCR master mix with the FAM flurophore and probe/primer pairs specific to *RASSF1A* (Applied Biosystems, Rockville, MD) according to the manufacturer’s cycling conditions using CFX96 thermal cyclers (Bio-Rad, Hercules, CA). Gene expression levels were normalized to the averages of expression levels of beta-actin and TATA-binding protein probe/primer pairs (Applied Biosystems, Rockville, MD). The Cycle Threshold (C_T_) values were calculated using the recommended Livak method (Bio-Rad, Hercules, CA).

### Methylation-specific PCR

Methylation status of CpG Island A of the *RASSF1A* promoter was assessed by MethylScreen technology using the Epitect methyl II PCR assay (Qiagen, Valencia, CA). Briefly, 125 ng of genomic DNA was mock-digested or digested with methylation-sensitive and methylation-dependent restriction enzymes individually or together, and the methylation status of the target sequence was measured using real-time qPCR with probes specific to the target promoter sequences. The amplification results that corresponds to >60% digestion by methylation-dependent restriction enzyme represents Hypermethylated sequences and 0% digestion indicates completely unmethylated DNA. Any amount of digestion between 0% and 60% represents the ‘intermediate methylation’ fraction. The Cycle Threshold (C_T_) values were converted into percentages of unmethylated, intermediately-methylated and hypermethylated CpG values, using a quantitation algorithm provided by the manufacturer (EpiTect Methyl II PCR Assay Handbook – Qiagen, Valencia, CA).

### Immunohistochemical (IHC) and Immunofluorescence (IF) detection

Five μm-thick FFPE sections were processed for immunohistochemistry according to the protocol recommended by the manufacturer of 3,3’Diaminobenzidine (DAB) substrate (BD Biosciences, San Jose, CA). Mouse anti-RASSF1A (1:100) primary antibody (Abcam, Cambridge, MA), goat anti-mouse/Biotin antibody (Santa Cruz Biotech., Santa Cruz, CA) and streptavidin-HRP (Life technologies, Rockville, MD) were used prior to DAB substrate development and detection (BD Biosciences, San Jose, CA). Nikon Eclipse E600 microscope with Spot 3.5 program was used to take photomicrographs at a total magnification of 400X. Immunofluorescence detection of RASSF1A proteins and microtubules were carried out as described [55]. Mouse anti-RASSF1A mAb (1:100; Abcam, Cambridge, MA) or goat anti-RASSF1A goat polyclonal (1:200; Santa CruZ Biotech, Santa Cruz, CA) primary antibodies and anti-goat FITC, anti-goat TR, anti-mouse FITC, or anti-mouse TR secondary antibodies (1:1000; all from Santa Cruz Biotech., Santa Cruz, CA) were used followed by ultracruz mounting agent containing 4’,6-diamidino-2-phenylindole - DAPI (Santa Cruz Biotech., Santa Cruz, CA) for immunodetection. Anti-DDK (DYKDDDDK epitope) monoclonal antibody (1:200; Origene, Rockville, MD) was used for detecting DDK-tagged *RASSF1A* and *RASSF1A/A133S* in transfected cells. Rhodamine Phalloidin, a high affinity F-actin probe conjugated to the red-orange fluorescent dye, tetramethylrhodamine (TRITC) (Biotium, Hayward, CA) was used for immunofluorescence detection of microtubules. Dilutions and incubations were carried out per the manufacturer’s recommendations. Zeiss AX10 confocal microscope with AxioVision 4.8 program was used for immunofluorescence analysis and photomicrographs were taken at a total magnification of 1000X.

### Cell culture, expression vectors, transfections, and Western blot detection

The human ACC cell line SW-13 was purchased from American Type Cell Collection (Manassas, VA) and was maintained under sterile conditions in DMEM supplemented with 10% certified fetal bovine serum and 10,000 U/mL penicillin/streptomycin (all from Life Technologies, Inc., Rockville, MD) in a standard humidified incubator at 37.0 C and 5% CO_2_. Myc-DDK tagged pCMV6-Entry, pCMV6-Entry/RASSF1A, and pCMC6-Entry/RASSF1A/A133S plasmid vectors (Origene, Rockville, MD) were used for transfection. Transfected SW-13 cells were designated SW-13/V representing pCMV vector alone, SW-13/A representing pCMV-RASSF1A, and SW-13/AM representing pCMV-RASSF1A/A133S mutant.

Transient transfection was carried out using Lipofectamine2000 according to the manufacturer’s recommendations (Life Technologies, Inc., Rockville, MD) in 6-well plates with a starting density of 80,000 cells/well. Transfected cells were allowed to grow for 6 days, to test the effect of *RASSF1A* and *RASSF1A/A133S* mutant gene expression on growth potential and survival of SW-13 cells. Total cell numbers and viability were calculated by staining cells with 0.4% Trypan Blue (GIBCO-BRL, Life Technologies, Inc., Rockville, MD) and manual counting using a counting chamber (Housser Scientific Co., PA). Experiments were performed in triplicate, and parallel plates with cells growing on glass coverslips were used to determine transfection efficiency and continued expression of transfected genes by indirect immunofluorescence.

Stable clones expressing RASSF1A and RASSF1A/A133S were selected in 800 μg/ml G-418 (Life technologies Inc., Rockville, MD) containing growth medium. Multiple clones were then pooled into populations to avoid expression variability between clones. Established populations (designated SW-13/V, SW-13/A, and SW-13/AM representing pCMV vector alone, pCMV-RASSF1A, and pCMV-RASSF1A/A133S mutant, respectively) were used to determine the effects of constitutive expression of RASSF1A or RASSF1A/A133S on SW-13 cell’s malignant behavior. Expression of transfected genes were confirmed via Western blotting using anti-DDK mAb for (1:1000; Origene, MD), anti-RASSF1A mAb (1:500, Abcam, MA), anti-mouse-HRP (Santa Cruz Biotech., CA), mini-PROTEAN TGX gel, PVDF blotting membrane (BioRad, Hercules, CA), and enhanced chemiluminescnce (ECL) detection reagents (Pierce Thermo Scientific, Rockford, IL) according to the manufacturer’s protocols. Equal protein loading between lanes were confirmed by staining PVDF membranes after chemiluminescence detection.

### Cell migration, invasion, and clonogenicity assays

Stable SW-13/V, SW-13/A or SW-13/AM cells were allowed to invade through a Matrigel layer from upper chambers containing serum-free medium to the lower chamber containing 10% FBS medium in BDBiocoat matrigel invasion chambers (BD Biosciences, Bedford, MA). After 24 hours, the Matrigel was removed, and invaded cells were fixed in 3.7% formaldehyde/PBS for 10 minutes, stained with 0.5% crystal violet for 2 hours, and counted using 10X magnification with a light microscope. The Matrigel invasion assay was performed twice in duplicate chambers. In the migration assay, the stably-transfected cells were allowed to migrate through 8 uM pore size modified Boyden Chambers (BD Biosciences, Bedford, MA) from upper chambers containing serum free medium to the lower chamber containing 10% FBS medium. After 4 hours, cells that migrated to the lower side of the membrane towards a higher FBS concentration gradient were fixed in 3.7% formaldehyde/PBS for 10 minutes, stained with 0.5% crystal violet and tabulated in triplicate. For clonogenicity assays, the cells were seeded in 6-well plates in low densities (5000 cells/well) and allowed to grow for 7 days in 400 μg/ml G-418 containing growth medium with a change of medium after 3 days. Cells were washed with PBS, fixed with 3.7% formaldehyde/PBS solution, stained with 0.5% crystal violet, and colonies with 10 +/− 2 cells were counted and averaged from 6 wells after performing the assay in quadruplicate.

### Statistical analysis

Significance of observed differences in sample means was evaluated using independent samples t-tests or ANOVA where appropriate after ensuring normality of distribution (Shapiro-Wilk test) and equivalence of variance (Levene’s test). *P-*values less than 0.05 were considered to be significant in all cases. Analysis was performed using SPSS v.19 (IBM Corporation, Armonk, NY).

## Competing interests

The authors declare that there are no competing interests that could be perceived as prejudicing the impartiality of the reported study.

## Authors’ contributions

T Carling and R Korah conceived, developed, designed and coordinated the study. R Korah performed gene expression studies, IF and IHC. J Healy and J W Kunstman conducted biochemical assays, statistical analyses, provided intellectual input and participated in the preparation of the manuscript. A Ameri participated in the cell culture work. A Fonseca participated in the procurement and processing of fresh-frozen and FFPE specimens. M Prasad provided histopathological expertise and critically revised the manuscript. All authors read and approved the final manuscript.

## Grant support

This work was supported by an Ohse Research Award. T.C. is a Doris Duke-Damon Runyon Clinical Investigator supported in part by the Damon Runyon Cancer Research Foundation and the Doris Duke Charitable Foundation.
